# High-Throughput Profiling of Colorectal Cancer Liver Metastases Reveals Intra- and Inter-Patient Heterogeneity in the EGFR and WNT Pathways Associated with Clinical Outcome

**DOI:** 10.3390/cancers14092084

**Published:** 2022-04-21

**Authors:** Kerstin Menck, Darius Wlochowitz, Astrid Wachter, Lena-Christin Conradi, Alexander Wolff, Andreas H. Scheel, Ulrike Korf, Stefan Wiemann, Hans-Ulrich Schildhaus, Hanibal Bohnenberger, Edgar Wingender, Tobias Pukrop, Kia Homayounfar, Tim Beißbarth, Annalen Bleckmann

**Affiliations:** 1 Department of Medicine A, Hematology, Oncology, and Pneumology, University Hospital Münster, 48149 Münster, Germany; kerstin.menck@ukmuenster.de; 2 West German Cancer Center, University Hospital Münster, 48149 Münster, Germany; 3 Department of Hematology/Medical Oncology, University Medical Center Göttingen, 37075 Göttingen, Germany; tobias.pukrop@ukr.de; 4 Department of Medical Bioinformatics, University Medical Center Göttingen, 37075 Göttingen, Germany; darius.wlochowitz@bioinf.med.uni-goettingen.de (D.W.); astrid.wachter@web.de (A.W.); alexander.wolff@med.uni-goettingen.de (A.W.); edgar.wingender@genexplain.com (E.W.); tim.beissbarth@bioinf.med.uni-goettingen.de (T.B.); 5 Department of General, Visceral and Pediatric Surgery, University Medical Center Göttingen, 37075 Göttingen, Germany; lena.conradi@med.uni-goettingen.de (L.-C.C.); kia.homayounfar@gnh.net (K.H.); 6 Department of Pathology, Universal Hospital of Köln, 50937 Köln, Germany; andreas.scheel@uk-koeln.de; 7 Division of Molecular Genome Analysis, German Cancer Research Center (DKFZ), 69120 Heidelberg, Germany; s.wiemann@dkfz-heidelberg.de; 8 Institute of Pathology, University Medical Center Göttingen, 37075 Göttingen, Germany; hans-ulrich.schildhaus@uk-essen.de (H.-U.S.); hanibal.bohnenberger@med.uni-goettingen.de (H.B.); 9 Clinic for Internal Medicine III, Hematology and Medical Oncology, University Regensburg, 93053 Regensburg, Germany

**Keywords:** colorectal cancer, liver metastasis, intratumoral heterogeneity, EGFR, WNT, high-throughput profiling

## Abstract

**Simple Summary:**

Tumor heterogeneity can greatly influence therapy outcome and patient survival. In this study, we aimed at unraveling inter- and intra-patient heterogeneity of colorectal cancer liver metastases (CRLM). To this end, we comprehensively characterized CRLM using state-of-the-art high-throughput technologies combined with bioinformatics analyses. We found a high degree of inter- and intra-patient heterogeneity among the metastases, in particular in genes of the WNT and EGFR pathways. Through analyzing the master regulators and effectors associated with the regulation of these genes, we identified a specific gene signature that was highly expressed in a large cohort of colorectal cancer patients and associated with clinical outcome.

**Abstract:**

Seventy percent of patients with colorectal cancer develop liver metastases (CRLM), which are a decisive factor in cancer progression. Therapy outcome is largely influenced by tumor heterogeneity, but the intra- and inter-patient heterogeneity of CRLM has been poorly studied. In particular, the contribution of the WNT and EGFR pathways, which are both frequently deregulated in colorectal cancer, has not yet been addressed in this context. To this end, we comprehensively characterized normal liver tissue and eight CRLM from two patients by standardized histopathological, molecular, and proteomic subtyping. Suitable fresh-frozen tissue samples were profiled by transcriptome sequencing (RNA-Seq) and proteomic profiling with reverse phase protein arrays (RPPA) combined with bioinformatic analyses to assess tumor heterogeneity and identify WNT- and EGFR-related master regulators and metastatic effectors. A standardized data analysis pipeline for integrating RNA-Seq with clinical, proteomic, and genetic data was established. Dimensionality reduction of the transcriptome data revealed a distinct signature for CRLM differing from normal liver tissue and indicated a high degree of tumor heterogeneity. WNT and EGFR signaling were highly active in CRLM and the genes of both pathways were heterogeneously expressed between the two patients as well as between the synchronous metastases of a single patient. An analysis of the master regulators and metastatic effectors implicated in the regulation of these genes revealed a set of four genes (SFN, IGF2BP1, STAT1, PIK3CG) that were differentially expressed in CRLM and were associated with clinical outcome in a large cohort of colorectal cancer patients as well as CRLM samples. In conclusion, high-throughput profiling enabled us to define a CRLM-specific signature and revealed the genes of the WNT and EGFR pathways associated with inter- and intra-patient heterogeneity, which were validated as prognostic biomarkers in CRC primary tumors as well as liver metastases.

## 1. Introduction

Colorectal cancer (CRC) is the third most common cancer in the more-developed regions worldwide [1]. Recent data from an autopsy study showed that approximately 70% of CRC patients will develop liver metastases (CRLM), a major cause of cancer-related death, during the course of their disease [2]. There is an interdisciplinary consensus that surgical resection of such metastases is the only treatment offering long-term survival and a potential cure, but, unfortunately, only one-third of patients are suitable for primary metastasis resection [3]. As shown by the CELIM trial, for example, secondary resectability can be achieved in an additional third by preoperative systemic chemotherapy including anti- epidermal growth factor receptor (EGFR) or anti-vascular endothelial growth factor (VEGF)-targeted therapy based on the individual *RAS* mutational status [4].

EGFR has been reported as one important factor involved in the development and progression of CRC [5]. Ligand binding induces receptor homodimerization and activation followed by signal transduction through Signal transducer and activator of transcription (STAT) proteins, Phosphatidylinositol 3-kinase (PI3K)-RAC-alpha serine/threonine-protein kinase (AKT), Mitogen-activated protein kinase (MAPK), and Proto-oncogene tyrosine-protein kinase Src (SRC) family kinases, which leads to increased cell proliferation, growth, and inhibition of apoptosis [6]. At the cellular level, *EGFR* mRNA expression was shown to be deregulated in CRLM compared to the primary tumor, and CRC cells overexpressing *EGFR* showed a metastatic phenotype [7,8], suggesting that *EGFR* plays an important role in CRLM.

EGFR signaling can crosstalk with the WNT pathway, which is considered the main molecular driver of CRC tumorigenesis. The binding of a canonical WNT ligand (e.g., WNT3A) to a receptor of the Frizzled (FZD) family and the co-receptor Low-density lipoprotein receptor-related protein 5/6 (LRP5/6) induces the release of β-catenin/CTNNB1 from the destruction complex comprised of AXIN, Glycogen synthase kinase-3 (GSK-3), Casein kinase 1 (CK1), and the tumor suppressor Adenomatous polyposis coli protein (APC). Subsequently, CTNNB1 translocates into the nucleus where it binds to transcription factors of the Transcription factor 7 (TCF)/Lymphoid enhancer-binding factor 1 (LEF) family and activates the expression of target genes involved in proliferation and differentiation. CRC patients often harbor mutations in *APC*, *CTNNB1*, or *RNF43*, which cause aberrant activation of the pathway and drive oncogenic transformation [9]. Alternatively, some WNT ligands (e.g., WNT5A) can also induce CTNNB1-independent, non-canonical signal transduction that mostly results in cytoskeletal rearrangements and increased cell motility and invasiveness [10]. Recent data suggest that both WNT subpathways are active in CRC cells [11].

Around 20% of CRC patients present with synchronous metastases at the time of diagnosis and 40% will develop metachronous metastases after resection of the primary tumor [12]. When comparing changes in gene expression in synchronous and metachronous metastases, the EGFR pathway was shown to be significantly upregulated in metachronous metastases, whereas processes related to angiogenesis were mainly affected in synchronous metastases [13]. The role of the WNT signaling pathway has not yet been investigated in this context, but the important role of the non-canonical WNT pathway has already been demonstrated in breast cancer metastasizing to the liver and brain [14,15,16]. Interestingly, in some patients with multiple CRLM, the metastases have been observed to react differentially to targeted chemotherapy. Furthermore, CRLM in a single patient share malignant features but also show heterogeneity and can differ in their mutational status of *KRAS*, *NRAS*, *BRAF*, or *PIK3CA* [17,18,19]. A high degree of intra-patient, inter-metastatic heterogeneity was shown to be associated with significantly shorter overall survival and is believed to arise from not only heterogeneity within the tumor itself, but also from clonal evolution [20]. Given that in the current clinical routine the decision for targeted therapy, e.g., the use of anti-EGFR antibodies, is commonly based on the *RAS*-mutational status of a single tumor biopsy, inter-metastatic heterogeneity is likely to greatly influence the treatment outcomes of CRLM.

Whole exome sequencing of brain metastases has revealed that genetic heterogeneity not only exists between metastases, but also between the primary tumor and its metastases [21]. Since most previous studies have focused on comparing the primary tumor with one single metastasis [22,23], gene and protein expression profiles of metachronous and synchronous metastases from individual patients are scarce but could give valuable information about the development of intra-patient heterogeneity, as well as identify possible drivers of tumor progression. In order to analyze inter-metastatic heterogeneity, we profiled and compared metachronous and synchronous liver metastases as well as normal liver tissue from two, clinically well-annotated CRC patients. There, we put our special focus on the two commonly deregulated pathways, EGFR and WNT. The samples were characterized at the gene and protein expression level by transcriptome sequencing and proteomic profiling via reverse phase protein arrays (RPPA), as well as by standardized histopathological and molecular subtyping. Using these means, we aimed at identifying master regulators that could drive metastasis as well as intra-patient heterogeneity.

## 2. Materials and Methods

### 2.1. Patients and Tissue Samples

**Figure 1:**
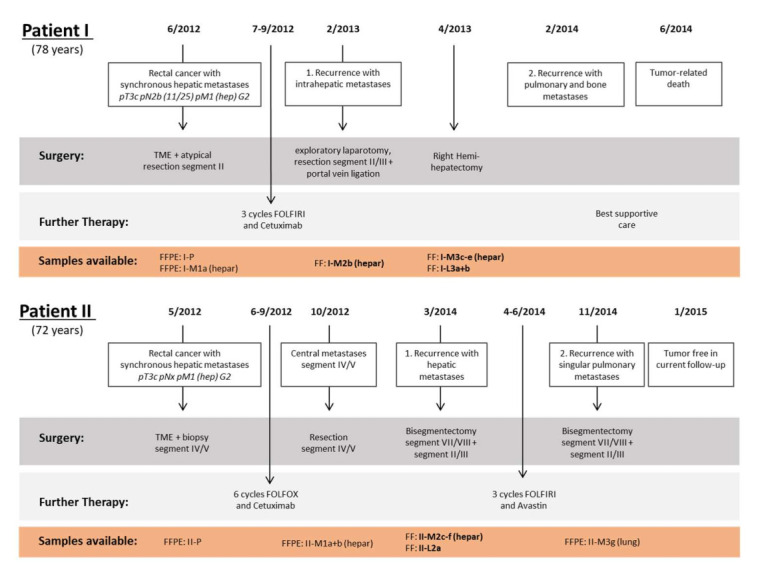
Timeline of patient treatment and course of the disease including obtained samples. Sequenced samples are displayed in bold.

For this pilot study, approved by the local ethics committee of the University of Medicine, Göttingen, Germany (21/3/11), and with the informed consent of the patients, tissue samples were collected from two patients with CRC and synchronous hepatic metastases. Samples comprised material from the primary tumor (P), from synchronous and metachronous metastases (M), as well as from normal liver tissue (L). Metastasis and normal liver tissue were stored as fresh-frozen (FF) and formalin-fixed and paraffin-embedded (FFPE) samples. The primary tumor tissue (P) had been stored as FFPE and was not used for RNA-Seq and RPPA profiling. The samples were coded using patient number (I or II), tissue type (P, M, or L), time point (1, 2, 3), and which metastasis (a, b, etc.). A detailed description of the samples is given in Figure 1.

#### 2.1.1. Patient I

Patient I was a 78-year-old male diagnosed in June 2012 with locally advanced rectal cancer and bilobar synchronous liver metastases involving segments II, IVa, V, and VI. Initial surgery consisted of low anterior resection with total mesorectal excision (TME) and resection of the superficial liver metastases in segment II (FFPE: I-P; FFPE: I-M1a (hepar)). Histopathological classification at this time was pT3c pN2b (11/25) pM1 (hepar) G2. The patient was then treated with three cycles of systemic chemotherapy with FOLFIRI and cetuximab. In February 2013, a relaparotomy was performed but the liver volume that would have remained after the planned extended right hemihepatectomy was deemed insufficient. Therefore, the liver metastasis in segment IVa was resected (FF: I-M2b (hepar)) and the right branch of the portal vein was ligated to induce contralateral liver hypertrophy. Two months later the left lobe had hypertrophied sufficiently to allow the secondary right hemihepatectomy. Histopathological analysis revealed three metastases (FF: I-M3c-e (hepar)). In addition, two samples of normal liver tissue were stored (FF: I-L3a + b). The patient remained tumor-free until February 2014, but died four months later due to tumor progression.

#### 2.1.2. Patient II

Patient II was a 72-year-old male who had been diagnosed with clinically non-metastasized rectal cancer in May 2012. A previously undetected solitary liver lesion was found in segment IV/V during the initial low anterior resection with TME. This lesion was identified as a metastasis by intraoperative biopsy. The initial tumor stage was thus pT3 pNx pM1 (hepar) (FFPE: II-P). The patient received systemic chemotherapy with FOLFOX and cetuximab. The follow-up computed tomography (CT) scan showed a good regression of the hepatic metastasis with no signs of extrahepatic disease. A non-anatomic liver resection was performed in October 2012. The histopathological workup showed two adenocarcinoma metastases (FFPE: II-M1a + b (hepar)). In March 2014, recurrent intrahepatic metastases were diagnosed. Bisegmentectomy of segment VII/VIII and non-anatomic resection of segments II and III revealed four metastases, which were resected R0 (FF: II-M2c-f (hepar)). In addition, a normal liver tissue sample was obtained (FF: II-L2a). Three cycles of systemic chemotherapy with FOLFIRI and bevacizumab were administered postoperatively. In November 2014, a singular intrapulmonary metastatic lesion was diagnosed and resected in toto (FFPE: II-M3g (lung)). The patient has been tumor-free since.

Expression of selected genes in a larger patient cohort was analyzed in normal colon tissue (*n* = 377), primary colon tumors (*n* = 1450), and colon cancer metastases (*n* = 99) on microarray data using the TNMplot database (https://www.tnmplot.com/, last accessed on 10 March 2022) [24]. The prognostic significance of the identified six MRs and MEs differentially expressed in both CRC patients was assessed in a cohort of rectal cancer patients (*n* = 165) from the Cancer Genome Atlas (TCGA) using the kmplot database (https://kmplot.com/, last accessed on 10 March 2022) [25].

### 2.2. Histopathological Assessment of Tissue Samples and Mutational Analysis

The patients were comprehensively characterized both clinically as well as by standardized histopathological and molecular subtyping prior to transcriptome sequencing. The method described by van Dam et al. was used to characterize the histopathological growth pattern of the individual metastases [26]. All samples were assessed by an experienced pathologist with regard to tumor cell content, amount of stroma, inflammatory infiltration, and necrosis. Only tumors containing >60% tumor cells were analyzed further. For mutation analysis, DNA was isolated from formalin-fixed tumor tissue followed by library preparation using a QIAseq targeted DNA custom panel. Target regions include *KRAS* exon 2–4, *NRAS* exon 2–4, *BRAF* exon 11 and 15, *PIK3CA* exon 2, 5, 6, 8, 10, and 21, and *TP53* exon 5–11. Next-generation sequencing was performed on an Illumina NextSeq instrument (San Diego, CA, USA) with subsequent data analysis on the CLC Genomics Workbench (Qiagen, Hilden, Germany).

### 2.3. Proteome Profiling

In order to specifically analyze EGFR and WNT signaling in normal and metastatic tissue, 110 antibodies against the core proteins of both pathways (Table S1) were selected. Samples of normal liver tissue and metastases were cryosectioned to obtain 10 µM slices. Tissue protein extraction reagent (T-PER, Pierce Biotechnology, Rockford, IL, USA) was complemented with 1 mM EDTA, 5 mM NaF, 2 μM staurosporine, PhosSTOP phosphatase inhibitor cocktail (Sigma-Aldrich, St. Louis, MO, USA), and complete mini protease inhibitor cocktail (Roche Diagnostics, Basel, Switzerland). Ice-cold tissue lysis buffer was added to each aliquot (10 µL buffer per 1 mg tumor) and thawed on ice for 5 min. A stainless-steel bead was added to each tube, and the samples were homogenized for 4 min at 30 Hz in a bead mill (Qiagen, Hilden, Germany). After lysis, the samples were placed on ice for 5 min and then placed on dry ice. The frozen tumor lysates were thawed on wet ice and centrifuged at 16,000× *g* for 10 min at 4 °C. The supernatants were transferred to homogenizer tubes (QIAshredder, Qiagen, Hilden, Germany) and centrifuged at 16,000× *g* for 1 min at 4 °C. Total protein concentration was determined using a modified bicinchoninic acid assay. The homogenized tumor lysates were aliquoted and stored at −80 °C. For further processing, the aliquots were thawed on wet ice and the total protein concentration was adjusted to 0.8 µg/µL. Prior to printing, the samples were mixed with 4× printing buffer (10% glycerol, 4% SDS, 10 mM DTT, 125 mM Tris, pH 6.8) and heated for 5 min at 95 °C. An amount of 24 µL of each sample was transferred to a 384-well plate and centrifuged for 2 min at 200× *g*. Samples were printed as technical triplicates in two identical subarrays on nitrocellulose-coated glass slides (Oncyte Avid, Grace Bio-Labs, Bend, OR, USA) using a contact printer (2470 Arrayer, Aushon Biosystems, Billerica, MA, USA) equipped with 185 µM solid pins employing 4 × 3 pins with 4.5 mm pin spacing. The humidity during the printing run was kept at 80%. The slides were stored afterward with desiccant at −20 °C. They were then mounted in 2-pad incubation chambers (Pepperprint, Heidelberg, Germany) and blocked for 2 h at room temperature with a modified fluorescent Western blotting blocking buffer (Rockland Immunochemicals, Limerick, PA, USA) mixed 1:1 with TBS (pH 7.6) containing 5 mM NaF, and 1 mM Na_3_VO_4_. Each subarray was subsequently probed overnight with primary antibody at 4 °C or incubated without primary antibody as blank control. The slides were washed four times for 5 min with TBST. Primary antibodies were detected with Alexa Fluor 680 F(ab’)2 fragments of goat anti-mouse IgG or anti-rabbit IgG at a dilution of 1:8000 for 1 h at room temperature. The slides were washed again in TBST (4 × 5 min) followed by a final rinse with ultrapure water for 5 min before air-drying. Separate slides were stained with Fast Green FCF for total protein quantification as described previously. The slides were scanned at an excitation wavelength of 685 nm and with a resolution of 21 µm using the Odyssey Infrared Imaging System (LI-COR Biosciences, Bad Homburg, Germany).

### 2.4. RNA Extraction and Gene Expression Analysis

For the transcriptome analyses, total RNA was extracted from FF material using Trizol (Thermo Fisher Scientific, Waltham, MA, USA) reagent. For this, ten 5 µm sections were cut at −20 °C under RNase-free conditions, and the extracted RNA was stored according to the manufacturer’s instructions (Life Technologies, Carlsbad, CA, USA). The RNA was resuspended in RNase-free water and stored at −80 °C. RNA integrity was assessed by microfluidic electrophoresis with the Agilent Bioanalyzer 2100 (Agilent Technologies, Santa Clara, CA, USA). Only samples with comparable RNA integrity numbers (RIN) greater than 7.0 were selected for deep sequencing. A 1 µg sample of total RNA was used as starting material for library preparation (TruSeq Stranded mRNA Sample Prep Kit, #RS-122-2101, Illumina, San Diego, CA, USA) for RNA sequencing (RNA-Seq). Accurate quantitation of cDNA libraries was performed with the QuantiFluor dsDNA System (Promega, Madison, WI, USA). The size range of cDNA libraries was determined using the DNA 1000 chip on the Bioanalyzer 2100 (280bp). The cDNA libraries were amplified and sequenced with the cBot and HiSeq 2000 from Illumina (SR, 1 × 51 bp, 8–9 gigabases > 40 Mio reads per sample). Sequence images were transformed with the Illumina software BaseCaller to bcl-files and then demultiplexed to FASTQ files with CASAVA (v1.8.2). Quality was checked using FastQC (v0.10.1, Babraham Bioinformatics). The RNA-Seq data have been uploaded to the GEO repository under the identifier GSE162960. The expression of selected genes was validated by quantitative real-time PCR (qRT-PCR). An amount of 1 µg of RNA was transcribed into cDNA with the iScript cDNA synthesis kit (Bio-Rad, Feldkirchen, Germany) and gene expression was measured from 10 ng cDNA at the QIAquant 384 5plex qRT-PCR system (Qiagen, Hilden, Germany) using SYBR green detection and primers listed in Table S2. For normalization, the housekeeping gene GNB2L1 was used.

### 2.5. Detection of Differentially Expressed Proteins and mRNAs

**Figure 2:**
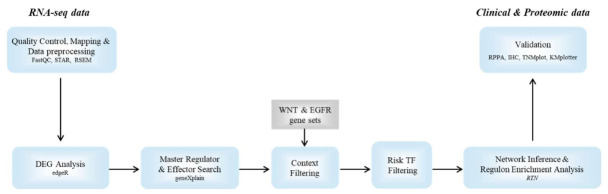
Analysis pipeline integrating RNA-Seq data with clinical and proteomic data from normal liver and metastatic tissues. RNA-Seq data was processed after initial quality check by mapping with STAR and counting with RSEM. Differential gene expression analysis was performed with ‘edgeR’. Differentially expressed genes (DEGs) were supplied to master regulator and effector workflows in the geneXplain platform (TRANSPATH) and candidates were filtered for their involvement in WNT and/or EGFR signaling. Risk transcription factors (TFs) associated with metastatic effectors and gene expression data were provided to network inference to create transcriptional regulatory networks (TRN). Regulon enrichment analysis with DEGs as input was performed to identify transcriptional drivers of metastasis, which were compared with available clinical data.

Bioinformatic analyses were conducted with the free statistical software R (v3.2.1; available from: www.r-project.org, accessed on 10 March 2022) (R Core Team. R: A language and environment for statistical computing. (2014). at http://www.r-project.org/, accessed on 10 March 2022). The RNA-Seq pipeline is illustrated as part of Figure 2. The quality check of FASTQ files yielded a GC content of 48–51%. Single-end 50-basepair reads were mapped against the Ensembl human reference genome GRC38.78 using STAR (v2.1.0a) [27]. The unique mapping length was 49.83 bp with a mapping rate of 84%. Counting was performed with RSEM (v1.2.19) [28], and the R package ‘edgeR’ (v3.8.6) [29] was used for differential expression analysis. P-values were adjusted for multiple testing with the method of Benjamini and Hochberg [30]. In downstream analysis, a false discovery rate (FDR) of up to 5% was considered significant. The principal component plot was generated with the R package ‘ggplot2’ (v2.0.0) [31]. Different gene sets comprising all significant genes were selected for the gene ontology (GO)-based enrichment analysis. The R package ‘topGO’ (v2.18.0) was used to detect significant levels of GO terms (*p* < 0.05) with the weighted Fisher’s exact test in the package. Gene set enrichment analysis (GSEA) was performed using the R package ‘clusterProfiler’ (v4.2.1) [32] to identify gene sets (≥20 genes) associated with the GO category biological process (BP) using the log2 fold changes obtained from differential expression analysis for each gene. GSEA interprets the degree of enrichment using normalized enrichment scores (NES) [33]. A positive NES indicates pathway activation, whereas a negative NES indicates pathway suppression. An adjusted *p* of <0.05 (Benjamini–Hochberg method) was used to define GO BP terms with significant enrichment. Enrichment results were visualized using the R package ‘enrichplot’ (v1.14.1). RPPA data were analyzed as described elsewhere [34,35]. Differential expression analysis at the protein level was performed with R package ‘limma’ (v3.26.9) [29]. Heatmaps were generated using correlation distance and complete linkage; normal tissue was used as the reference for protein levels.

### 2.6. Estimating Intratumor Heterogeneity (ITH)

Genes with zero read counts across all samples (8 CRLM and 3 normal liver controls) in the RSEM read count data were removed before analysis. RSEM normalized counts were then obtained by dividing each read count by the 75th percentile of the read counts in its sample multiplied by a factor of 1000. The DEPTH algorithm in the R package ‘DEPTH’ (v1.0) was used to estimate the tumor heterogeneity level of each metastatic sample based on log2 transformed RSEM normalized data [36].

### 2.7. EGFR and WNT Pathway-Related Gene Collections and WNT Pathway Overrepresentation Analysis

An EGFR pathway-related gene collection was generated from TRANSPATH^®^ 2013.4 [37] by merging the “EGF pathway” (TRANSPATH^®^ ID: CH000000722) and the “ErbB3 ⟶ survival” pathway (TRANSPATH^®^ ID: CH000004191) within the geneXplain platform, resulting in a list of 164 gene symbols (Table S3). WNT pathway-related gene collections were generated as described in [38] and are provided in Table S4. Canonical and non-canonical WNT gene sets were further tested for overrepresentation of differentially expressed proteins using the Wilcoxon rank sum test. If antibodies reacted with multiple proteins of the same family, only one corresponding gene ID was used.

### 2.8. Search for Master Regulators (MRs) and Metastatic Effectors (MEs) in WNT and EGFR Signaling Pathways

Master regulators (MRs) and metastatic effectors (MEs) were searched for upstream and downstream, respectively, of the discovered differentially expressed genes (DEGs), utilizing analysis workflows of the geneXplain platform (https://genexplain.com/, accessed on 10 March 2022). MRs are defined as molecules that sit at the top of the regulatory hierarchy in signal transduction pathways and potentially orchestrate the changes in gene expression observed at several levels or steps. In contrast, MEs are defined as molecules that sit at the very bottom of the regulatory hierarchy and, thus, their activity is modulated by any of the upstream molecules. For a set of genes of interest, MRs and MEs are searched for by applying a modified shortest-path algorithm that explores the graph for possible common regulators or key nodes using the pathway knowledge of the TRANSPATH^®^ database. The MRs’ core algorithm has been previously described in [39,40], whereas an inverted version of this algorithm is applied in the case of the MEs. In our analyses, we used the MR and ME search workflow (each with FDR < 0.05) called ‘Find master regulators in networks (TRANSPATH^®^)’ and ‘Find effectors in networks (TRANSPATH^®^)’, of the geneXplain platform web edition 6.2 (https://genexplain.gwdg.de/bioumlweb/, last accessed on 22 June 2021). Workflows were run with a maximum search radius of 10 steps upstream and downstream of significantly up- and downregulated DEGs (|log2FC| > 2, FDR < 0.05) to obtain significant MRs and MEs.

### 2.9. Network Inference and Regulon Enrichment Analysis

We performed network inference to investigate the regulatory relationships between risk transcription factors (TFs) included in the list of MEs and their potential target genes. To this end, an integrative network-based approach was applied to the gene expression data of CRLM to reveal putative transcriptional drivers of the DEGs discovered in the patients’ pairwise comparison. Prior to network inference, RSEM read count data was pre-filtered to exclude genes, where there are less than four samples with read counts greater than or equal to 10. The read count data were then normalized using the regularized-logarithm transformation (rlog) function in the R package ‘DESeq2′ (v1.32.0) with option blind set as ‘True’ [41].

The normalized gene expression data and a list of risk TFs (MEs in EGFR and WNT pathway-related gene collections) were provided as inputs to the R package ‘RTN’ (v2.16.0) [42,43] with default options. Briefly, the RTN algorithm assesses the statistical dependence between gene expression data and TFs for the reconstruction of transcriptional regulatory networks (TRNs). Regulatory units (regulons) consisting of TF-target pairs are inferred using the ARACNe algorithm [44], which is re-implemented in the RTN package. Furthermore, the regulatory relationship (positive or negative) in a TF-target pair is inferred using Spearman’s correlation. Prior to applying the ARACNe algorithm, RTN performs permutation (1000 permutations, BH-adjusted *p*-value < 0.01) to eliminate non-significant TF-target pairs, followed by bootstrapping (100 bootstraps, 95% consensus) and the data-processing inequality filter (eps = 0) to eliminate unstable TF-target pairs and indirect TF-target pairs, respectively. The R package ‘RedeR’ (v1.40.0) [45] was used to visualize the TRN. Finally, we applied the MR analysis implemented in RTN with default options to assess whether the observed regulons are enriched for the DEGs (|log2FC| > 2, FDR < 0.05) in the patients’ pairwise comparison, filtering down our initial list of risk TFs to potential metastasis-relevant TFs.

### 2.10. Survival Analyses

Overall survival (OS) analyses were conducted for 43 patients with CRLM (*n* = 51). Prior to analysis, normalized gene expression (rlog, ‘DESeq2′) was averaged over samples related to the same patient. High/low groups were created based on gene expression levels for single genes (*GNAO1*, *IGF2BP1*, *PIK3CG*, *PRKCB*, *SFN*, *SMAD3*, and *STAT1*) by using an optimal cutoff value determined using the surv_cutpoint function in the R package ‘survminer’ (v0.4.8). The log rank test was used to compare different survival rates between the groups using Kaplan–Meier analysis. The R package ‘survival’ was used to calculate P-values and hazard ratios (HR).

### 2.11. Immunohistochemistry

Immunohistochemical staining to assess microsatellite instability (MSI) and TP53 expression was performed on FFPE tissue samples as described previously [46]. Briefly, tissue sections cut into 2 μM-thick slices were incubated in EnVision Flex Target Retrieval Solution, pH high (Dako/Agilent, Santa Clara, CA, USA) followed by incubation of primary antibodies against MutL homolog 1 (MLH1) (#IR079), MutS Homolog 2 (MSH2) (#IR085), MutS Homolog 6 (MSH6) (#IR086), PMS1 Homolog 2, Mismatch Repair System Component (PMS2) (#IR08761), and tumor protein p53 (TP53) (#GA61661, all from Dako/Agilent, Santa Clara, CA, USA) at room temperature for 20 min. To visualize the sites of immunoprecipitations, secondary antibodies coupled to HRPO peroxidase (EnVision Flex+) and DAB (both from Dako/Agilent, Santa Clara, CA, USA) were applied, and stainings were evaluated by light microscopy after counterstaining with Meyer’s haematoxylin. For immunohistochemical staining of SMAD3, heat epitope retrieval was performed for 60 min at 100 °C followed by incubation with the SMAD3 antibody (#25494-1-AP, Proteintech, Planegg-Martinsried, Germany) for 36 min after preconditioning with CC1 for 31 min. The OptiView DAB IHC Detection Kit (Ventana Medical Systems, Oro Valley, AZ, USA) was used as a secondary antibody. The slides were screened at a low magnification for the pattern and distribution of the staining.

## 3. Results

### 3.1. The Metastases Did Not Differ in Their Histopathological Growth Patterns in the Individual Patients

Both patients were comprehensively characterized histopathologically and by molecular subtyping (Table 1). All primaries and CRLM were shown to be KRAS, NRAS, BRAF, and PIK3CA wild-type. The CRLM of both patients showed no signs of microsatellite instability (MSI), but displayed an overexpression of TP53 (Figure S1), which was associated with the inactivating TP53 Arg273His mutation in patient I. The samples that were included in RNA-Seq and RPPA profiling consisted of at least 60% vital tumor cells with no more than 40% necrosis. The growth pattern analysis allocated the metastases of patient I to the aggressive replacement type and that of patient II to the more favorable desmoplastic type, which corresponds well with the clinical outcome in both patients. Of note, no marked difference in histopathological subtype was seen between the metastases of each patient.

### 3.2. Healthy Liver Tissue of Both Patients Exhibited a Higher Degree of Similarity Than the Metastases from an Individual Patient

**Figure 3:**
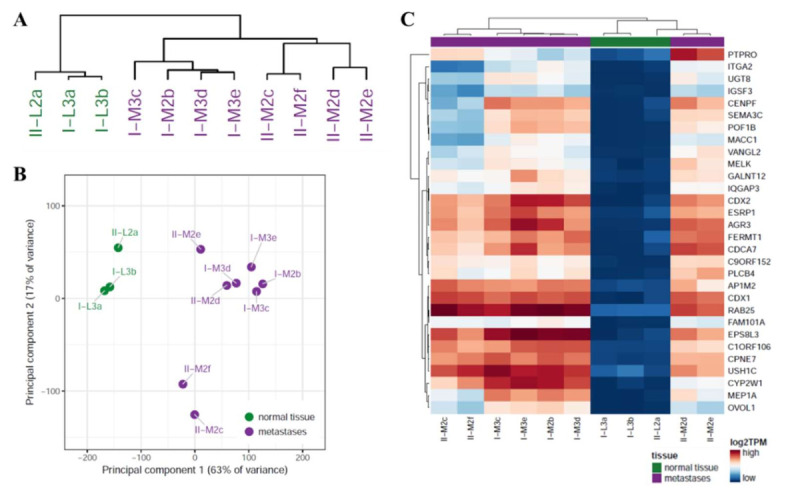
Gene expression signature of CRLM compared with normal liver tissue. (**A**) Complete linkage-based dendrogram of all measured transcripts comprising normal liver samples (green) and CRLM of patients I and II (purple). (**B**) Principal component analysis of normal liver tissue and CRLM samples from patients I and II. (**C**) Heatmap displaying log2 transcripts per million (TPM) of the top 30 transcripts differentially expressed in normal liver (green) and CRLM (purple). Metastatic drivers include CRC markers such as *CDX1* and *CDX2*, and WNT-pathway genes (*VANGL2*, *PLCB4*).

To compare the degree of heterogeneity of the CRLM with that of normal liver tissue, we characterized the gene expression patterns by RNA-Seq. Unsupervised, hierarchical clustering of the data revealed a greater similarity between the metastatic samples of both patients compared to the respective adjacent normal liver. Of note, the correlation between the normal liver tissue samples from the two patients was stronger than the correlation between the different metastatic samples from an individual patient, thus hinting at a greater heterogeneity within the metastases (Figure 3A). The principal component analysis confirmed large differences between the normal liver and metastatic samples yet separated two metastatic samples (II-M2f and II-M2c) from the other CRLM (Figure 3B). We cannot exclude that this separation might be attributed to different sequencing batches, and batch information was therefore taken into account for subsequent differential gene expression analyses. The comparison of the normal liver tissue with the metastatic samples resulted in 3287 significant (FDR < 0.05) DEGs (Table S5). GO term enrichment analysis was performed to pinpoint the differences between CRLM and normal liver tissue on a functional level. In line with the known high metabolic activity of hepatocytes, the analysis revealed that particular pathways related to metabolism were among the most significant GO terms (Figure S2). This tissue-specific difference was confirmed by the analysis of the top 30 DEGs (Figure 3C), which comprised the two well-studied intestinal transcription factors *CDX1* and *CDX2* that were absent in normal liver tissue but highly expressed in all CRLM samples. In line with the histopathological characterization (Table 1), this observation supports the assumption that the gene expression pattern of the metastatic tissue is indeed largely attributable to intestinal CRC cells. Other transcripts present at high levels in all CRLM were the WNT pathway activator *FERMT1*, the cell cycle protein *CDCA7*, and the pro-metastatic RNA-binding protein *ESRP1* [47,48,49], which underlines the malignant phenotype of the metastasized CRC cells. *RAB25*, which has been described as acting primarily as a tumor suppressor in CRC primary tumors, but as an oncogene in other cancer subtypes [50], was present at high levels in all metastatic samples of patients I and II.

As both the WNT and the EGFR signaling pathways are known to be highly active in primary CRC tumors, we next asked whether the same held true for CRLM. Therefore, we focused our analysis on DEGs associated with either of the pathways (Figure S3). In total, 12 genes of the EGFR pathway and 110 genes of the WNT pathway displayed significantly different expression patterns in CRLM compared with normal liver tissue, and most of them were highly upregulated in CRLM. With regard to the WNT pathway, the top 30 DEGs comprised several genes associated with CTNNB1-independent signaling (e.g., *CAMK2B*, *VANGL2*), however, the majority of DEGs were known target genes of the classical CTNNB1-dependent, canonical pathway (e.g., *AXIN2*, *LGR5*, *TCF7*, *CDX1*), which is consistent with its known role as a driver of tumorigenesis in CRC. Interestingly, with regard to EGFR signaling the DEG analysis demonstrated an upregulation of several associated intracellular kinases (e.g., *SRC*, *MAPK3*, *PIK3C2B*), whereas the differences in the *EGFR* expression itself were rather minimal. Taken together, these observations revealed a distinct gene expression signature of CRLM, which includes known drivers of CRC progression as well as WNT and EGFR signaling, and clearly separates the CRLM from normal liver tissue.

### 3.3. The Metastases of Patient I Displayed a Higher Degree of Intra-Tumoral Heterogeneity

In order to further analyze the intra-tumoral heterogeneity of the CRLM in relation to the normal liver tissue, we calculated the individual DEPTH scores (Table 2). This score is a measure of the deviation in the gene expression compared to the mean gene expression values of the normal tissue and has been shown to correlate well with genomic instability, immunosuppression, and unfavorable tumor phenotypes [36]. In line with this, MSI and mutation of the tumor suppressor gene *TP53* are associated with higher DEPTH scores.

Indeed, all metastases from the *TP53*-mutated patient I displayed consistently higher DEPTH scores than the metastases from patient II (median patient I: 12.3, median patient II: 8.42), with the exception of metastasis II-M2c. The analysis furthermore revealed largely different DEPTH scores among the metastases from one individual patient, pinpointing a high degree of inter-metastatic heterogeneity. In comparison, melanoma as one of the cancer types with the highest tumor mutational burden (TMB) was shown to display a median DEPTH score of 17.73, whereas prostate adenocarcinomas with low TMB possessed a median DEPTH score of 2.95 [36].

**Figure 4:**
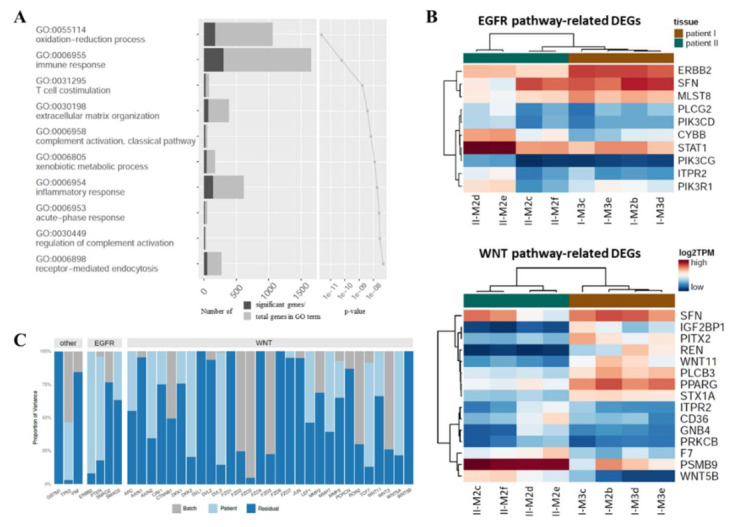
Inter- and intra-patient heterogeneity of metastases. (**A**) Results of GO term enrichment analysis showing the main differences between the metastases of the two patients with regard to immune response, inflammatory response, and metabolic processes. Listed are the top ten significant GO terms. (**B**) Heatmaps displaying log2 transcripts per million (TPM) of the top differentially expressed transcripts comparing patient I (brown) against patient II (green). The upper panel shows all differentially expressed genes related to EGFR signaling, the lower panel shows the top 15 differentially expressed genes related to WNT signaling. (**C**) Variance component analysis of metastases for selected transcripts of interest.

When we compared the DEGs of the metastases of the two patients, the GO term enrichment analysis revealed immune response and inflammatory and oxidation-reduction processes to be among the top ten significant GO terms (Figure 4A, Table S6). Likewise, GSEA confirmed the enrichment of pathways associated with metabolism, the immune system, and development (Figure S4A, Table S6). These tissue differences cannot be attributed solely to different tumor cell contents, but also reflect general tissue differences between the patients and could point to different immune reactions within the tumor of the individual patients, as already suggested by the DEPTH score.

The analysis of single DEGs in the metastases of the two patients revealed 2,254 genes with significantly differential expression (Table S7), among them the DNA repair gene *MGMT* as one of the most significant DEGs that was lost in all metastases of patient I. As *MGMT* is important for maintaining the integrity of the genome, its loss could contribute to the higher genomic instability observed in this patient. Interestingly, the metastases in patient I were further characterized by a significant downregulation of *PTPRO*, a negative regulator of EGFR signaling that is associated with poor prognosis in CRC patients [51,52]. Taken together, the data suggested a high degree of inter-metastatic heterogeneity in CRLM with a possible link to activated EGFR signaling in poor-outcome patient I.

### 3.4. WNT and EGFR Genes Are Associated with Inter-Patient and Inter-Metastatic Heterogeneity

Since the gene expression analyses had suggested deregulation of the EGFR as well as the WNT pathways, which both play pivotal roles in CRC, we focused on the expression pattern of genes related to both pathways in the CRLM of the two patients (Figure 4B). A total of 10 genes of the EGFR pathway and 61 genes of the WNT pathway were found to be differentially expressed between the two patients. With regard to the WNT gene expression pattern, the metastases of each patient clustered together, although sample I-M2b had been resected two months prior to the other metastases in patient I. This suggested large inter-patient, rather than inter-metastatic, expression differences in WNT genes. In contrast, the expression of EGFR-related genes revealed larger inter-metastatic differences, as the two metastases II-M2c and II-M2f from patient II exhibited a greater similarity with the metastases of patient I than with the other two metastases of patient II.

A closer analysis of the WNT-related DEGs suggested that, in particular, CTNNB1-independent WNT signaling was differentially activated in the CRLM of both patients since the two non-canonical WNT ligands, *WNT11* and *WNT5B*, were identified among the most significant DEGs (Figure 4B). Both have already been linked to the invasive properties of CRC cells and poor patient survival [53,54,55]. Intriguingly, all CRLM of patient II (favorable outcome) were characterized by a strikingly high expression of the WNT pathway-related immunoproteasome gene *PSMB9*, which has been associated with enhanced lymphocyte infiltration and longer survival in breast cancer patients [56].

To determine the influence of patient-specific differences, proportional contributions of different effects that can be attributed to the variance in gene expression were evaluated. Therefore, a variance component analysis of selected genes from the EGFR and WNT pathways was performed (Figure 4C). Variance contributions from batches, patients, and residual components were discriminated; the latter being expected to include inter-metastatic effects. In the set of selected EGFR pathway-related genes, particularly *SMAD2* and *SMAD3* showed large residual effects, which implies a link to TGFB1 signaling. In the selected set of WNT pathway-related genes, the key components *AXIN1*, *DVL1*, and *DVL2* as well as the WNT receptors *FZD1*, *FZD4*, *FZD6*, and *FZD7* showed large residual effects suggesting large inter-metastatic differences. This also applied to *PORCN*, which is crucial for WNT secretion, as well as *JUN* and *WNT5B*, which are associated with activation of non-canonical WNT signaling. Of note, we also found a high residual effect for *GSTM1* and *VIM*, two genes known to support tumor progression and metastasis. In contrast, great patient–effect contributions were found for *ERBB2* and *PTEN* among the EGFR pathway-related genes and for *DKK2*, *DVL3*, *TCF7*, and *WNT5A* among the WNT pathway-related genes. Taken together, this approach identified several genes of the WNT and EGFR pathways that contribute to the observed inter-patient as well as inter-metastatic differences.

### 3.5. EGFR and WNT Signaling Are Active in CRLM, in Particular in Poor-Prognosis Patient I

**Figure 5:**
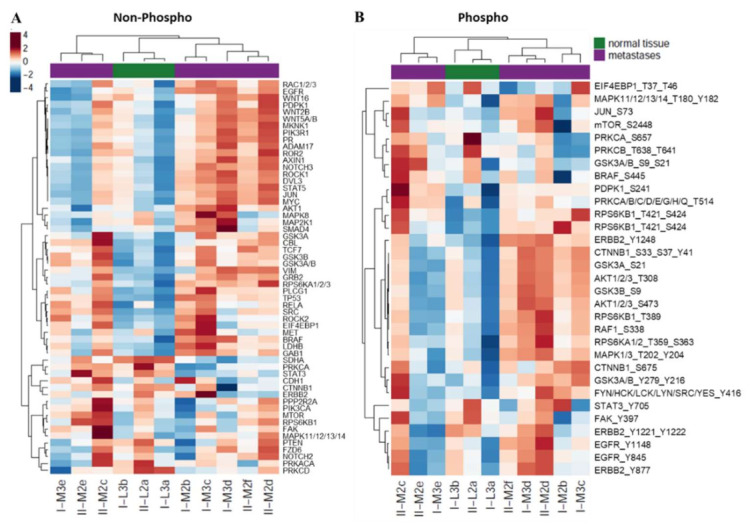
RPPA data reveal high inter-metastatic heterogeneity in WNT- and EGFR-related proteins, but clearly separate CRLM from normal liver. (**A**,**B**) Normal liver samples (green) and CRLM from both patients (purple) were characterized by RPPA for the expression of total proteins (**A**) and phosphorylated proteins (**B**) associated with either the WNT or the EGFR signaling pathway. Protein levels were normalized to the median of the normal tissue samples.

In both the EGFR as well as the WNT pathways, extracellular signals are transmitted through intracellular downstream kinases activating distinct signaling responses. In order to assess whether the observed gene expression changes in the metastases of the two patients were mirrored at the protein level, we characterized the normal liver and CRLM samples by RPPA. As in the results from the RNA-Seq, the normal tissue samples from both patients clustered together and were clearly distinct from the metastases (Figure 5A,B, Figure S4B). Out of the 93 tested proteins, 27 were significantly (*p* < 0.05) differentially expressed (Table S8). With the exception of the two samples I-M3e and II-M2e, the proteins of both pathways, WNT and EGFR, seemed to be rather highly-expressed at the total protein level as well as in their respective phosphorylated forms compared to the normal liver tissue. Sample II-M2c showed a strikingly elevated expression in all the investigated total and phosphorylated proteins. Of note, the same sample had displayed the highest DEPTH score among the CRLM of patient II, suggesting that it differs from the other synchronous metastases of the patient at both the transcriptomic and proteomic levels.

Testing for the enrichment of proteins associated with either the non-canonical or the canonical WNT pathway revealed a significant (*p* = 0.047) or nearly significant (*p* = 0.059) overrepresentation, respectively. Again, this indicated that not only classical canonical but also non-canonical WNT signaling plays a role in CRLM. Compared with the gene expression analysis, RPPA profiling revealed a much greater heterogeneity in many of the tested proteins in synchronously occurring metastases from the same patient (e.g., for I-M3c-e or II-M2c-f). Vimentin (VIM) was more highly expressed in CRLM than in normal liver tissue, which fits well with its role as a biomarker for epithelial-to-mesenchymal transition (EMT), an essential step in successful metastasis. Furthermore, a particularly strong upregulation of the tyrosine kinase SRC, which is associated with active EGFR as well as WNT signaling, was detected in the metastases (Table S8). In parallel, the protein Succinate Dehydrogenase Complex Flavoprotein Subunit A (SDHA) was significantly downregulated in the metastatic tissue. SDHA has recently been shown to inhibit canonical WNT signaling, and consequently the proliferation and invasion of cancer cells [57], suggesting that the loss of SDHA in CRLM could be linked to hyperactive WNT signaling. Fostering this hypothesis, PRKCD (PKCδ) and PRKCA (PKCα), two negative modulators of canonical WNT signaling [58,59], were among the most significantly downregulated proteins in CRLM. Enhanced activity of PRKCA has been shown to inhibit the transcriptional activity of CTNNB1 and induce apoptosis in CRC cells [59]. PRKCA was not only differentially expressed in CRLM compared with the healthy liver but also when the differentially expressed proteins in the CRLM of both patients were compared (Figure 5A,B, Table S9). The metastases of poor-outcome patient I displayed particularly low levels of both the total PRKCA protein as well as its active forms with phosphorylation at S657, T638, and T641, again suggesting hyperactive WNT signaling with enhanced proliferation and invasion in the metastases of this patient. The comparison further identified higher levels of phosphorylated (S9, S21), and thus inactivated, GSK3 in patient I which would not support the hypothesis of a particularly strong activation of the WNT pathway. However, this result was only observed with one antibody, whereas the other two showed strong signals in the metastases of both patients, thus arguing against a reproducible effect. In summary, both pathways, WNT and EGFR, seemed to be active in CRLM, whereas the data point toward a particularly strong activity of canonical WNT signaling in poor-outcome patient I.

### 3.6. WNT- and EGFR-Associated Master Regulators and Metastatic Effectors of CRLM Are Highly Upregulated in a Large Cohort of CRC Patients

**Figure 6:**
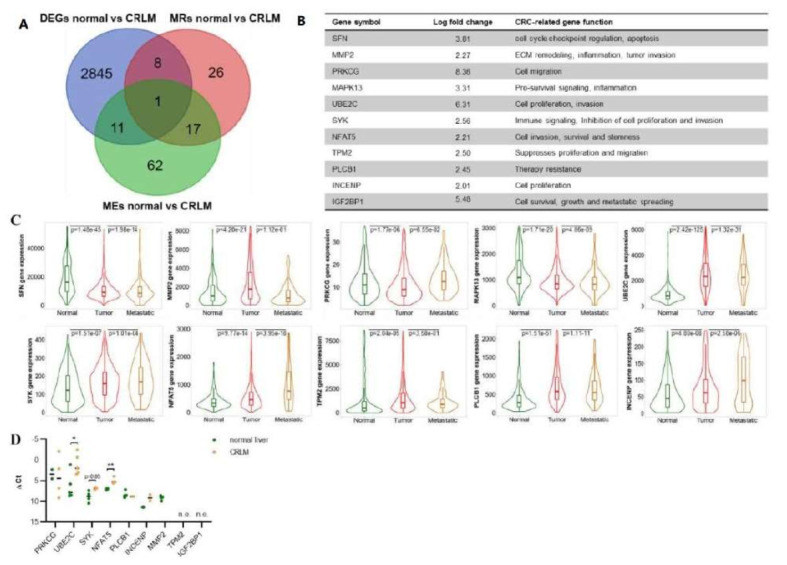
Master regulators and metastatic effectors implicated in CRLM are upregulated in a large cohort of patients with metastatic colon cancer. (**A**,**B**) Comparison of the gene expression pattern of normal liver tissue and CRLM: VENN diagram (**A**) depicting the overlap of the DEGs with the identified WNT- and EGFR-related master regulators (MRs) and metastatic effectors (MEs). The MRs and MEs that were identified among the significant DEGs and displayed a |log2 fold change| >2 are listed in (**B**) with an annotation of their known function and significance in CRC. (**C**) Expression of the identified MRs and MEs was analyzed in normal tissue (*n* = 377), primary colon tumors (*n* = 1450), and colon cancer metastases (*n* = 99) using the TNMplot database (TNMplot.com). Significance was calculated with a Dunn’s test. No data were available for IGF2BP1. (D) Expression of the indicated genes was analyzed in matched CRLM and normal liver samples from five CRC patients by qRT-PCR (line: median, * *p* < 0.05, ** *p* < 0.01, n.e.: not expressed). Significance was calculated with a two-sided *t*-test. Missing values relate to absent expression of certain genes in some patients.

We next aimed at identifying common master regulators and effectors associated with CRLM. To this end, active signal transduction pathways were studied on the basis of prior knowledge of signaling pathways to determine the regulatory link between gene expression and gene abundance. First, upstream master regulators (MRs) and downstream effectors (MEs) of significantly up- and downregulated DEGs (|log2FC| > 2, FDR < 0.05) were identified based on the comparison of the healthy liver vs. CRLM. To study the relevance of WNT and EGFR signaling in this context, we restricted the identified MRs and MEs to genes that have been associated with one of the pathways. This gave 52 MRs and 91 MEs (Table S10). To confirm the results, we aligned these two gene sets with the genes that were included in the input list of DEGs (normal liver vs. CRLM) for the MR and ME analyses (Figure 6A,B). The resulting 11 genes, which showed a significantly strong upregulation in CRLM (|log2FC| > 2, FDR < 0.05) and have potential relevance in the formation of CRLM, were then analyzed for their expression in normal colon tissue, primary colon tumors, and colon cancer metastases in a larger patient cohort using the TNMplot database [24] (Figure 6C). 

With the exception of *SFN* and *MAPK13*, all other genes were upregulated in colon cancer primary tumors compared with normal colon tissue and/or in colon cancer metastases compared with the primary tumor. To further confirm these findings, we measured the expression of the remaining 9 genes in matched samples of CRLM and normal liver tissue from five CRC patients by qRT-PCR (Figure 6D). Although *TPM2* and *IGF2BP1* expression was not detectable in these patients, there was a significant upregulation of *UBE2C* and *NFAT5* in CRLM compared to the normal liver as well as a trend for *SYK* and *INCENP*. This implies that our approach had indeed identified a set of MRs and MEs, which are highly expressed in a large number of patients and can be linked to tumor growth and metastatic spread in colorectal cancer.

### 3.7. Analysis of Gene Regulatory Networks Identifies WNT- and EGFR-Associated Master Regulators and Metastatic Effectors Associated with Poor Survival in CRC

**Figure 7:**
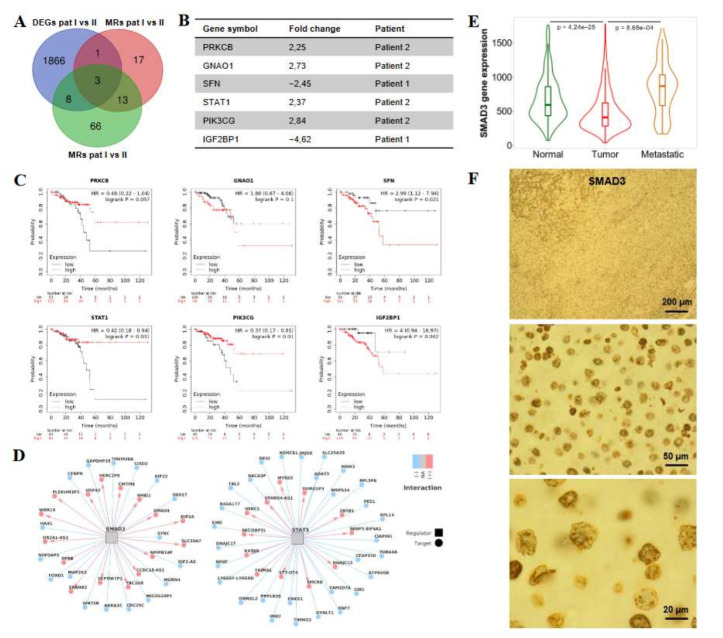
SMAD3 is a master regulator in the metastases of poor-outcome patient I. (**A**,**B**) Comparison of the gene expression patterns of patients I and II: VENN diagram (**A**) depicting the overlap of the DEGs with the identified WNT- and EGFR-related master regulators (MRs) and metastatic effectors (MEs). The MRs and MEs that were identified among the significant DEGs and displayed a |log2 fold change| > 2 are listed in (**B**) and the enrichment in the respective patient is indicated. (**C**) Kaplan–Meier plots depicting overall survival of rectal cancer patients (*n* = 165) depending on the expression of the identified MRs and MEs. The data were obtained from the Kaplan–Meier plotter database (kmplot.com). (**D**) Transcriptional regulatory networks of the two identified regulons inferred by ARACNe. Edges in blue: positive regulatory relationship in TF-target pair; edges in red: negative regulatory relationship in TF-target pair. (**E**) Expression of SMAD3 in normal tissue (*n* = 377), primary colon tumors (*n* = 1450), and colon cancer metastases (*n* = 99) was analyzed using the TNMplot database (TNMplot.com). Significance was calculated with a Dunn’s test. (**F**) IHC staining of SMAD3 expression in the metastases of patient I at different magnifications.

As both patients in our study differed in their clinical outcome, we were interested in identifying genes associated with inter-patient heterogeneity that might explain the difference in tumor aggressiveness. Thus, the analysis of WNT- and EGFR-related MRs and MEs was performed as explained above based on the comparison of the CRLM of patients I and patient II (Table S11). This resulted in 12 MRs and MEs that were significantly differentially expressed between both patients, out of which six displayed a |log2FC| > 2 (Figure 7 A,B). Interestingly, *SFN* and *IGF2BP2*, which were more highly expressed in the metastases of poor-outcome patient I, were also associated with shorter overall survival in a large cohort (*n* = 165) of rectal cancer patients based on data from the Kaplan–Meier plotter database [25]. In contrast, high expression of *STAT1* and *PIK3CG*, which were upregulated in the metastases of patient II, was associated with a more favorable outcome (Figure 7C). Likewise, the same trend was observed for *PRKCB* (*p* = 0.057). No clear effect of *GNAO1* expression was observed on patient survival. To confirm the prognostic relevance of the identified MR and ME genes in a second independent dataset, we performed an RNA-Seq of 43 CRLM and correlated the expression level of the six genes with patient survival. The results confirmed that high expression of *SFN* and *IGF2BP2* was linked to poor outcome, whereas high expression of *STAT1* and *PIK3CG* was associated with prolonged survival (Figure S5). No significant difference in patient survival was observed for *PRKCB* or *GNAO1*.

Several of the discovered WNT- and EGFR-associated MEs corresponded to transcription factors (TFs) (*CEBPB*, *CTNNB1*, *ELK1*, *EP300*, *FOS*, *FOXO4*, *HDAC1*, *MITF*, *NFATC1*, *PPARD*, *PPARG*, *RXRA*, *SMAD2*, *SMAD3*, *SP1*, *STAT1*, *STAT3*) that could act as transcriptional drivers of processes relevant for CRLM. To further investigate the regulatory relationships between these 17 risk TFs and their potential target genes, we reconstructed regulatory units (regulons) consisting of TF-target pairs inferred by the ARACNe algorithm [44]. As a result, only two significant and stable regulons were obtained for *SMAD3* and *STAT3* (Figure 7D). We then performed an enrichment analysis to assess whether the observed regulons are enriched for the DEGs observed in CRLM of both patients and found significant enrichment for *SMAD3* (adj. *p* = 0.017), but not for *STAT3* (adj. *p* = 1). *SMAD3*, a downstream transcription factor of the TGFB1 pathway, was also significantly overexpressed in a large number of metastases from colon cancer patients compared with the primary tumors based on the TNMplot database (Figure 7E); however, it was downregulated when the primary tumor was compared with normal tissue. This context-dependent expression of *SMAD3* fits well with its established dual role as a tumor suppressor in early cancer and a tumor promoter in late-stage tumors in which it supports invasion and metastasis [60]. As the selected antibody panel in the RPPA measurements did not target SMAD3, immunohistochemical staining of the patients’ metastatic samples was performed to validate the expression of SMAD3 also in poor-outcome patient I. Indeed, SMAD3 was highly upregulated in patient I at the protein levels, although this was not the case at the level of gene expression (Figure 7F). Taken together, our data provide valuable insight into the gene regulatory networks present in CRLM and identify several candidate genes with a potential role in the formation and aggressiveness of CRLM.

## 4. Discussion

Cancers are known to become increasingly heterogeneous during the course of the disease. As a result, the genetic and phenotypic makeup of metastases tends to differ from the primary tumor and can lead to therapy failure and poor clinical outcome. However, inter- and intra-patient heterogeneity in the context of CRLM has so far been poorly investigated. In this study, we addressed this issue by comprehensively characterizing eight synchronous and metachronous CRLM from two CRC patients using high-throughput profiling to decipher their transcriptional and proteomic landscape. Our analysis identified a CRLM-specific signature that clearly discriminated metastatic samples from normal liver tissue and revealed a high degree of tumor heterogeneity in the genes of the EGFR and the WNT pathways, which have previously been associated with poor survival in CRC. 

A comparison of the general gene expression profile of CRLM and normal liver tissue suggested a greater inter-patient similarity in gene expression in the metastases of the two patients than between samples of normal liver and metastases of the same patients. This pattern has also been observed previously [22,23] and implies a common molecular metastatic profile. Many of the DEGs belonged to the WNT pathway, the main molecular driver of CRC tumorigenesis. Considering that the expression of common WNT-negative regulators (e.g., *SDHA*, *PRKCD*, *PRKCA*) was lost in CRLM, while at the same time activators (e.g., *FERMT1*, *VANGL2*, *SRC*) were highly upregulated, this implies that WNT signaling is hyperactive not only in primary CRC but also in CRLM. SRC was overexpressed in CRLM both at the transcriptional as well as the protein level. This corresponds to earlier observations in mouse models [61]. SRC is a tyrosine kinase with proto-oncogene characteristics that is well-characterized in CRC [62] and that can enhance nuclear translocation and the transcriptional activity of CTNNB1 [63]. There is evidence that high expression of SRC is associated with poor clinical outcome, and initial studies showed a role of non-receptor tyrosine kinases of the SRC family (e.g., SFK) in later steps of CRC as well, even if it is not yet well understood how they act in metastasis formation [64].

We furthermore identified *LGR5*, *TCF7*, *CDX1*, and *AXIN2* among the top WNT pathway-related genes enriched in CRLM. All of them are target genes of CTNNB1-dependent, canonical WNT signaling. In particular, *AXIN2* has been described as a strong tumor promoter for CRC in vivo by inducing EMT [65]. Interestingly, the data also pointed towards an activation of CTNNB1-independent WNT signaling in CRLM. Although non-canonical WNT signaling has long been neglected in CRC, it was recently shown that WNT ligands can activate both canonical as well as non-canonical WNT signaling in this tumor entity [11]. Although there seems to be a general trend toward the enrichment of non-canonical WNT proteins in CRLM, high inter-patient heterogeneity was observed for its two ligands, *WNT5B* and *WNT11*, and high inter-metastatic heterogeneity for several of the associated FZD receptors. As WNT downstream signaling is known to depend on the ligand–receptor–coreceptor combination, this suggests that different signaling responses could be induced in the individual patients and the individual metastases after ligand stimulation due to this difference in receptor status.

In order to identify upstream regulators and downstream effectors that could explain the differences in gene expression between normal liver tissue and CRLM, we performed an analysis of potential MRs and MEs which identified a set of 11 genes that were significantly enriched in CRLM. Of these genes, eight (*MMP2*, *PRKCG*, *UBE2C*, *SYK*, *NFAT5*, *TPM2*, *PLCB1*, *INCENP*) were found to be upregulated in a large patient cohort with primary and/or metastatic colon cancer and four (*UBE2C*, *NFAT5*, *SYK*, *INCENP*) could be further validated by qRT-PCR in CRLM samples. These genes require further functional validation as they could represent promising effectors involved in CRC development and progression.

Inter-patient analyses were performed to pinpoint tumor heterogeneity between the two CRC patients and analyze its relevance to their clinical outcomes. By comparing the metastases of patients I and II, genes involved in metabolic processes, inflammatory response, and extracellular matrix organization were found to be discriminative. Cancer cell metabolism is strongly associated with tumor and metastasis formation [66,67], and exploiting metabolic vulnerabilities has been discussed as a treatment strategy [68]. Immune-related processes could be influenced by chemotherapy but might also be linked to general discrepancies in the immune system of the two patients or to different antigenic responses to the tumor. In line with the latter, a comparison of the specific WNT pathway-related DEGs identified *PSMB9* as the most highly-expressed gene in CRLM of favorable-outcome patient II. In breast cancer, high levels of such immunoproteasome genes have been correlated with the abundance of tumor-infiltrating lymphocytes and a favorable prognosis [56]. Although the histopathological assessment had not revealed any notable differences in the immune infiltrates in the metastases of the two patients in our study, this finding could support the hypothesis of a stronger immune evasion phenotype in the metastases of patient I contributing to the poorer clinical outcome. This idea is supported by the higher DEPTH score observed in the CRLM of patient I, which is not only a measure for tumor heterogeneity and genomic instability but also for immunosuppression [36].

A high degree of heterogeneity in primary CRC has been shown to be associated with metastatic spread and shorter disease-free survival [69]. Another study analyzed intra-patient inter-metastatic heterogeneity in 134 CRLM samples from 45 CRC patients and found it to be of strong prognostic relevance [20]. Our study likewise revealed a high degree of intra-patient inter-metastatic heterogeneity in CRLM mirrored by the differences in DEPTH scores and RPPA profiles between the metastases from each individual patient. Moreover, the CRLM of patient I not only displayed a higher degree of heterogeneity, potentially caused by the detected inactivating *TP53* mutation and loss of *MGMT* expression, but were characterized by a concordant loss of WNT- and EGFR-negative regulators (e.g., *PTPRO*, *SFN*, *PRKCA*) [51,52,59,70] as well as a gain of WNT effectors (e.g., *IGF2BP1*) [71] which might have fostered hyperactive WNT/EGFR signaling and caused the poor clinical outcome. To gain insight into the underlying gene regulatory networks, we calculated the MRs and MEs based on the DEGs between both patients. Of note, genes associated with poor survival (*SFN*, *IGF2BP1*) were highly expressed in patient I, whereas genes associated with longer survival (*PRKCB*, *STAT1*, *PIK3CG*) were enriched in patient II. *SFN* and *IGF2BP1* are both associated with cell survival. In contrast, upregulation of *STAT1* and *PRKCB* has been linked to favorable outcome in CRC [72,73]. Likewise, an IHC study demonstrated a downregulation of *PIK3CG* in 85% of human CRC patients that was associated with increased invasion and metastasis [74].

Focusing on transcription factors responsible for the differential expression pattern of the identified MRs and MEs, we identified *SMAD3* as the main MR and confirmed its expression in the CRLM of patient I by IHC. *SMAD3* showed high inter-metastatic, but also high inter-patient heterogeneity. It is known as an important downstream transcription factor of the TGFB1 pathway and can directly interact with CTNNB1. This interaction protects CTNNB1 from degradation and enhances its nuclear translocation [75], thereby synergistically promoting CRC progression with the WNT pathway. Again, this might point to a particularly strong canonical WNT activity in the CRLM of poor-outcome patient I. It must be mentioned that TGFB1 can act both as a tumor suppressor and a tumor promoter, depending on the cellular context. In late-stage cancer, cells seem to become resistant to its anti-mitotic effects and TGFB1 was instead observed to stimulate EMT by upregulating mesenchymal markers (e.g., *VIM*, *CDH2*) and downregulating epithelial markers (e.g., *CDH1*) [60]. In line with this, in our RPPA analyses VIM was enriched in CRLM compared with normal liver tissue.

## 5. Conclusions

Taken together, our analyses have revealed a high degree of tumor heterogeneity at several levels: (1) between the normal liver tissue and CRLM, (2) between the CRLM of the two patients, and (3) between the individual CRLM from each patient. Genes of the WNT and EGFR pathway were identified as contributors to this heterogeneity, and although more mechanistic studies are needed to validate the underlying molecular mechanisms, the results suggest that strong WNT activity, genomic instability, and an immune evasion phenotype are associated with the poor outcome in patient I. Finally, based on the high-throughput profiling and bioinformatic analysis of the CRLM of both patients, we were able to identify *SFN* and *IGF2BP1* as genes that were upregulated in CRC metastasis and associated with poor survival in two independent cohorts of CRC patients, whereas *STAT1* and *PIK3CG* indicated a more favorable prognosis.

## Figures and Tables

**Table 1:** Histopathological characterization of metastatic samples.

Sample	Inflammatory Infiltrate (%)	Stroma (%)	Tumor (%)	Necrosis (%)	Growth Pattern
I-M2b	10	20	70	25	n.a.
I-M3c	10	30	60	40	Replacement
I-M3d	10	20	70	10	Replacement
I-M3e	10	20	70	5	Replacement
II-M2c	10	10	80	10	Desmoplastic
II-M2d	10	10	80	0	Desmoplastic
II-M2e	10	20	70	0	Desmoplastic
II-M2f	15	20	65	0	Desmoplastic

n.a. = not available.

**Table 2:** Intra-metastatic heterogeneity measured by DEPTH score.

Sample	Inflammatory Infiltrate (%)
I-M3b	14.43
I-M3c	14.07
I-M3d	9.03
I-M3e	10.39
II-M2c	11.43
II-M2d	8.21
II-M2e	6.92
II-M2f	8.62

## Data Availability

The RNA-Seq data have been uploaded to the GEO repository under the identifier GSE162960. All other data supporting the findings of this study are available within the article and the Supplementary Materials or from the corresponding author upon reasonable request.

## Supplementary Materials

The following are available online at www.mdpi.com/article/10.3390/cancers14092084/s1, Table S1: List of antibodies used for RPPA measurements; Table S2: List of primers used for qRT-PCR; Table S3: EGFR pathway-related gene set; Table S4: WNT pathway-related gene set; Table S5; Results of differential gene expression analysis between samples of normal liver and CRLM; Table S6: Significant biological process GO terms identified in enrichment analysis between CRLM of patients I and II; Table S7: Results of differential gene expression analysis between CRLM of patients I and II; Table S8: Results of differential protein abundance analysis by RPPA between normal liver and CRLM samples; Table S9: Results of differential protein abundance analysis by RPPA between CRLM of patient I and patient II; Table S10: Results of the MR and ME analysis for the comparison of normal liver versus CRLM; Table S11: Results of the MR and ME analysis for the comparison CRLM of patient 1 versus patient 2; Figure S1: Expression of mismatch repair proteins and TP53 in CRLM from both patients; Figure S2: GO term enrichment results comparing normal against metastatic tissue based on RNAseq data; Figure S3: Pathway-specific heatmaps displaying log2 transcripts per million (TPM) of the top differentially expressed transcripts comparing normal tissue against metastatic tissue; Figure S4: Principal component analysis of normal liver and metastatic tissue of patients I and II based on protein measurements.
